# Enhancing Hydrocracking Catalyst Performance and Lifetime through Surfactant-Templated Mesoporosity in Pt/HUSY Zeolites

**DOI:** 10.1021/acs.energyfuels.5c05175

**Published:** 2025-12-27

**Authors:** N. F. L. de Paula, H. M. Mesa, J. M. Ortigosa, M. A. S. Garcia, J. M. A. R. de Almeida, J. Garcia-Martinez, P. N. Romano

**Affiliations:** † Instituto de Química, 28125 Universidade Federal do Rio de Janeiro, Av. Athos da Silveira Ramos, 149, Rio de Janeiro 21941-909, Brazil; ‡ Laboratorio de Nanotecnología Molecular, Departamento de Química Inorgánica, 16718 Universidad de Alicante, Alicante 03690, Spain; § Nanotechnology Engineering Program, Alberto Luiz Coimbra Institute for Graduate Studies and Research in Engineering, COPPE, UFRJ, Rio de Janeiro 21941-972, Brazil; ∥ Programa de Pós-Graduação em Química (PGQu), UFRJ, Rio de Janeiro 21941-909, Brazil

## Abstract

The development of catalysts capable of overcoming mass transport limitations remains a crucial challenge in hydrocracking processes, particularly for bulky linear hydrocarbons, such as long-chain paraffins. In this study, we report the synthesis of hierarchical Pt/HUSY catalysts via a surfactant-templating strategy that introduces highly interconnected intracrystalline mesoporosity while preserving most of the zeolite’s crystallinity and acidity, significantly enhancing molecular diffusion, facilitating better access to acid sites, and alleviating the diffusion limitations typically associated with purely microporous zeolites. Catalytic testing revealed a marked increase in hexadecane cracking activity, especially in catalysts with longer surfactant treatment times (Pt/CBV780_20h and Pt/CBV720_41h), which exhibited superior conversion rates and selectivities toward lighter hydrocarbons. Notably, the surfactant-templated catalysts achieved similar product distributions at lower temperatures than those of their parent materials. Additionally, our study demonstrates the importance of well-connected intracrystalline mesoporosity in reducing catalyst deactivation by preventing coke accumulation, particularly in high-aluminum Pt/CBV720 catalysts, which showed higher stability over time despite greater coke deposition. These findings highlight the effectiveness of surfactant-templated mesoporosity as a powerful strategy to enhance both the performance and lifetime of zeolite-based catalysts for industrial hydrocarbon conversion processes.

## Graphical abstract

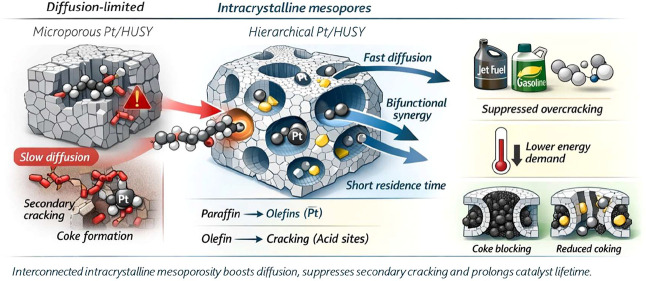

## 1 Introduction

The design of advanced catalytic materials capable of overcoming fundamental mass transport limitations remains a persistent challenge in heterogeneous catalysis, particularly for processes involving bulky hydrocarbon molecules such as long-chain paraffins.^1–3^ This challenge has significant industrial relevance in hydrocracking operations, where the transformation of heavy alkanes, commonly found in heavy gas oils, waxes, and vacuum gas oils, into valuable lighter fractions, such as gasoline, jet fuel, and diesel, is a core objective.^4^ In such systems, the inherent microporous architecture of conventional zeolites severely restricts the diffusion of these bulky molecules, limiting the accessibility of internal acid sites and leading to reduced catalytic efficiency, suboptimal product selectivity, and accelerated deactivation due to coke accumulation.^5,6^

Conventional zeolites, particularly ultrastable Y (USY), are widely used owing to their strong Bro̷nsted acidity, high thermal stability, and well-defined microporosity. However, the intrinsic micropore size imposes severe diffusional constraints that restrict the accessibility of the active sites.^7^ To address these limitations, hierarchical zeolites that integrate micropores with tailored mesoporosity have emerged as a promising strategy.^8–10^ In this context, hierarchical architectures can significantly enhance molecular transport, increase active site utilization, and improve resistance to coke formation. A common strategy to introduce mesoporosity into zeolites is controlled steaming and acid treatment; however, this typically generates a random, poorly interconnected pore network and often compromises the crystallinity, acidity, and structural integrity of zeolites.^11–13^

Among the various postsynthetic strategies developed to produce hierarchical zeolites, the surfactant-templated method stands out for its ability to introduce well-defined intracrystalline mesoporosity. This approach relies on the self-assembly of surfactant molecules into micelles, which serve as soft templates that lead to zeolite framework rearrangement, causing the formation of mesopores during the hydrothermal treatment. This process enables the formation of extensive intracrystalline mesoporosity with tunable size and high interconnectivity while preserving the essential properties of the zeolite, such as crystallinity, acidity, and thermal stability.^14^ A direct consequence of this is the emergence of an organized and accessible mesopore network that enhances the transport of large molecules into and out of zeolites. In addition to enhancing the molecular transport and improving access to the internal pore structure, engineered mesopore networks mitigate catalyst deactivation by reducing the risk of pore blockage and facilitating the removal of undesired species and coke precursors.^15,16^ Building on these advantages, the incorporation of noble metal nanoparticles (NPs), such as platinum, into hierarchical zeolites plays a pivotal role in enhancing their catalytic performance.^17–19^ Through their dehydrogenation–hydrogenation functionality, Pt NPs act synergistically with Bro̷nsted acid sites to promote paraffin-to-olefin conversion, followed by selective cracking.^17,18^ The catalytic efficiency strongly depends on the spatial proximity and balance between the metallic and acidic functions, ensuring rapid hydrogen transfer while minimizing secondary cracking. Tailoring the zeolite topology has proven to be particularly effective in strengthening this synergy during the hydroisomerization of *n*-hexadecane.^20–22^ Introducing intergrown or hierarchical frameworks that combine one-dimensional channels with mesoporous regions mitigates diffusion constraints by creating shorter and less tortuous pathways for long-chain *n*-paraffins.^20^ These features facilitate the faster migration of intermediates between the Pt and Bro̷nsted acid sites, accelerating the hydrogenation–isomerization sequence. Comparable improvements have also been observed in three-dimensional hierarchical systems incorporating mesostructured alumina or siliceous components, which enhance molecular accessibility, acid-site utilization, and metal dispersion.^21,22^ The resulting architectures, when combined with well-balanced acid density and strength, enable efficient coupling of hydrogenation and isomerization steps, achieving conversions exceeding 90% with excellent selectivity toward monobranched i-C_16_ isomers.

In addition to hydroisomerization, the influence of zeolite topology on C–C bond cleavage has been examined in hydrocracking reactions.^23^ In systems combining Y zeolites with Al-substituted mesoporous silica, the integration of micro- and mesoporous domains enhances diffusion and site accessibility. Pure Y zeolite exhibited moderate conversion, whereas the addition of a mesostructured siliceous component markedly increased activity and selectivity. Complementary to the diffusion effect, the presence of Al within the siliceous mesoporous framework introduced moderate-to-low Bro̷nsted acidity, favoring catalyst performance. The resulting material achieved higher conversion and superior selectivity toward midrange (C_5_–C_12_) hydrocarbons in contrast to the parent Y zeolite, which favored the formation of lighter cracking products. Similar enhancements occur in composite structures featuring mesoporous aluminosilicate shells surrounding microporous zeolitic cores, which also improve the hydrothermal stability and maintain high catalytic performance.^24^ Introducing mesoporosity through controlled structural rearrangement significantly influences catalytic performance and product distribution during the transformation of long-chain *n*-paraffins. In the desilication treatments, the framework of the parent zeolite was partially leached, generating a network of random mesopores that increased the overall pore volume and mesopore surface area.^25,26^ In Beta-based catalysts, this modification enhances the diffusional mobility of reactants and intermediates, yielding up to 83% *n*-hexadecane conversion compared to 63% for the unmodified zeolite.^25^ Despite these improvements, desilication partially disrupts the crystalline framework, leading to uncontrolled silicon extraction and alterations in the acid-site distribution. This effect is reflected by a pronounced increase in weak acid sites, from 48 to 141 μmol·g^–1^, without significant changes in the concentration of medium-to-strong sites, suggesting redistribution rather than total loss of acidity. The presence of these weaker sites may contribute to the primary transformations that precede the C–C bond cleavage.^23^ In Ni-supported Beta zeolites, partial desilication has also been employed to generate intracrystalline mesopores, resulting in a 31% increase in n-hexadecane conversion and a 12% reduction in coke formation relative to the parent Ni/H-Beta catalyst.^25^ These improvements arise from the alleviation of diffusional restrictions and formation of more easily removable carbonaceous species. Nonetheless, the partial loss of crystallinity and framework silicon highlights a key limitation of desilication-based methods, reinforcing the need for alternative approaches that introduce mesoporosity without compromising the structural integrity.

Therefore, the development of strategies that introduce mesoporosity while preserving the long-range order and intrinsic acidity of zeolites is a key step forward. Such approaches can deliver enhanced diffusion, improved accessibility, and greater stability without sacrificing the crystalline framework, which is essential for durable catalytic performance.

In this study, we provide a structure–function analysis of surfactant-templated Pt/HUSY catalysts for *n*-hexadecane hydrocracking. By systematically varying the surfactant-treatment time, we correlate mesopore formation with changes in acid-site accessibility and catalytic behavior. Unlike previous work that examined mesoporosity without directly linking it to hydrocracking performance, our results show that controlled mesostructuring leads to higher conversion, increased light-hydrocarbon yields, and improved stability. The combination of Pt with surfactant-templated USY zeolites enables a quantitative connection between mesoporosity, acid-site distribution, and hydrocracking performance. These findings demonstrate the central role of intracrystalline mesopores in enhancing catalytic efficiency and lifetime and provide clear guidance for designing next-generation hydrocracking catalysts.

## 2 Methods

### 2.1 Chemicals and Materials

Commercial CBV780 and CBV720 zeolites were obtained from Zeolyst International and used as the catalyst supports. All other organic and inorganic reagents were of analytical grade and were used without further purification. Ammonium hydroxide (NH_4_OH, 28–30%), cetyltrimethylammonium bromide (CTAB, >96%), ammonium nitrate (NH_4_NO_3_, >98%), tetraammineplatinum­(II) chloride hydrate (Pt­(NH_3_)_4_Cl_2_·*x*H_2_O, 98%), and hexadecane (ReagentPlus, 99%) were purchased from Sigma-Aldrich.

### 2.2 Surfactant Treatment

Surfactant-templated zeolites were synthesized using a postsynthetic treatment method as described previously.^14–17,27^ In a typical procedure, 2.5 g of zeolite (CBV780 or CBV720) was suspended in 160 mL of NH_4_OH solution (1.4 wt %), followed by the addition of 1.75 g of CTAB. For the CBV780 series, the mixture was magnetically stirred at 40 °C for either 3 or 20 h, the resulting samples were designated as CBV780_3h and CBV780_20h, based on the treatment duration. For the CBV720 series, after initial mixing, the suspension was stirred for 20 min at 40 °C and then subjected to hydrothermal treatment at 150 °C under static conditions for 3 or 41 h. The resulting materials were labeled CBV720_3h and CBV720_41h, respectively. After the respective treatments, all samples were thoroughly washed with distilled water until the pH of the washings approached neutral, then dried at 110 °C overnight. Subsequently, the materials were calcined at 550 °C for 6 h.

### 2.3 Preparation of Pt-Loaded Catalysts and Pretreatment

The Pt NPs were immobilized on the zeolites before utilization, enabling the hydrogenation of olefins produced from hexadecane cracking. An aqueous solution of 0.21 mol/L Pt­(NH_3_)_4_Cl_2_·*x*H_2_O was used. Using an incipient wetness impregnation procedure, the solution was added dropwise to each pretreated zeolite using a quantity of metal precursor to achieve 1.0 wt % Pt. After this process, the impregnated material was dried again at 120 °C overnight to remove residual moisture. Subsequently, mild calcination was performed at 400 °C for 12 h, at a heating rate of 5 °C/min.

### 2.4 Characterization Methods

The crystallinity of the samples was evaluated by X-ray diffraction (XRD) using a Bruker AXS D8 Advance diffractometer equipped with a Cu Kα radiation source (λ = 1.5406 Å), and monochromatized using a graphite crystal operating at 40 kV and 40 mA. Textural properties, including pore volume and pore size distribution, were determined from nitrogen adsorption–desorption isotherms measured at −196 °C using an AUTOSORB-6 surface area and porosity analyzer. Before analysis, the samples were degassed under vacuum (5 × 10^–5^ bar) at 250 °C for 8 h. The data were processed using the QuadraWin software (version 6.0, Quantachrome Instruments). The cumulative pore volumes and pore size distributions were calculated using the nonlocal density functional theory (NLDFT) model based on the adsorption branch. The total pore volume was obtained from the plateau of the cumulative adsorption curve at a relative pressure (*P*/*P*_0_) of 0.9. The mesopore volume was calculated by subtracting the micropore volume from total pore volume.^27^

The morphology, particle size, and spatial distribution of the Pt NPs on the zeolites before and after utilization were investigated using a JEOL JEM-2100 Plus Transmission Electron Microscope (TEM) operating at an accelerating voltage of 200 kV. For the sample preparation, a small quantity of each catalyst was dispersed in isopropyl alcohol and ultrasonicated for 15 min to ensure homogeneous dispersion. A drop of the resulting suspension was deposited onto a copper grid coated with carbon film for TEM analysis. The elemental compositions of the samples were analyzed by energy-dispersive X-ray spectroscopy (EDX) coupled with scanning electron microscopy (SEM) using a JEOL IT500HR/LA instrument to determine the Si/Al ratio of the framework.

The concentration of the acid sites in the materials was characterized by pyridine adsorption/desorption infrared spectroscopy (Py-FTIR) using a Thermo Scientific IS50-FT-IR spectrometer. Self-supporting wafers (∼1.2 cm diameter) were prepared from the catalyst powder and placed in a specialized IR cell connected to a vacuum line. The wafers were pretreated at 400 °C for 1 h under nitrogen flow (100 mL/min), followed by evacuation under vacuum for 3 h to eliminate physisorbed species. Pyridine vapor was introduced at 50 °C until equilibrium (∼600 Pa) was reached, followed by heating to 250 °C at a ramp rate of 5 °C/min and a subsequent 10 min vacuum treatment. Infrared spectra were recorded in the region of 1700–1400 cm^–1^ to identify the characteristic absorption bands of the acid sites. Quantification of the Bro̷nsted and Lewis acid sites was performed by integrating the bands at 1540 and 1450 cm^–1^, respectively, according to established procedures.^1^ Ammonia temperature-programmed desorption (NH_3_-TPD) was performed to evaluate the acidity of the samples to corroborate the Py-FTIR results. Prior to the measurements, each sample was pretreated under a helium flow at 400 °C to remove physisorbed species. The temperature was then lowered to 100 °C, followed by NH_3_ dosing, until surface saturation was achieved. After purging the physisorbed ammonia with helium, the desorption step was carried out by heating the sample from 100 °C to the final temperature at a ramp rate of 20 °C/min under a continuous helium flow. The resulting NH_3_ desorption profile was recorded to assess the strength distribution of acid sites.

Carbon monoxide (CO) chemisorption was employed to estimate the metal dispersion of the zeolite-supported catalysts. Before analysis, the samples were pretreated under hydrogen flow at 400 °C for 6 h, followed by cooling to room temperature. After signal stabilization, calibrated pulses of CO were introduced and the uptake was monitored using a mass spectrometer.

After the cracking reactions, coke formation was evaluated by thermogravimetric analysis (TG). The samples were exposed to an O_2_ atmosphere at a heating rate of 20 °C/min up to 800 °C, and the mass change was recorded as a function of time and temperature.

### 2.5 Catalytic Reaction and Products Assessment

The catalytic cracking reactions were carried out in an automated fixed-bed reactor system operating under continuous flow conditions. In a typical experiment, the reactor was prepared as follows: First, silicon carbide (SiC) was placed at the bottom of the reactor to prevent catalyst displacement. An additional layer of SiC was positioned above the catalyst bed to promote uniform radial distribution of the reactant. For the Pt/CBV780_Parent material, 200 mg of the catalyst was positioned between the SiC layers and glass wool films were used as separators. The catalyst mass was adjusted to provide nearly equivalent total Bro̷nsted acid sites (BAS) under identical reaction conditions. Because the CBV720 samples required substantially smaller catalyst masses to achieve BAS equivalence, they were diluted with inert glass beads such that the packed-bed height and void fraction matched those of the CBV780 beds. This ensured comparable bed hydrodynamics and, consequently, a constant residence time for the reacting species across all catalysts. The corresponding W/F values and additional experimental details are provided in Supporting Information (Table S1). Before the reaction, each catalyst underwent in situ activation by flowing hydrogen at 30 mL/min and 400 °C, maintaining these conditions for 4 h. After activation, the reactor was adjusted to the target reaction temperature and a pressure of 20 bar. Once the set temperature and pressure were reached, hexadecane was introduced at a flow rate of 0.03 mL/min, maintaining a hydrogen flow of 30 mL/min. A 4.5 h stabilization period was allowed to achieve steady-state conditions, followed by a 1 h product collection phase. Finally, at the end of the reaction, the reactor was purged to remove any residual reactants and then cooled to room temperature.

The reaction products were obtained in both the liquid and gaseous phases. The liquid fraction was collected in glass vials for subsequent dissolution and analysis, and the gaseous products were analyzed online. Both phases were quantitatively and qualitatively analyzed in a Shimadzu gas chromatographer GC-2030 equipped with a flame ionization detector in a DB-1 capillary column (100 m × 0.25 mm × 0.50 μm). Gas-phase chromatographic analyses showed no detectable C_1_–C_2_ hydrocarbons (CH_4_, C_2_H_6_, C_2_H_4_) under the applied reaction conditions. The identified products included C_3_–C_12_ hydrocarbons and hexadecane and its isomers. Hexadecane conversion was quantified based on the relative decrease in the chromatographic signal corresponding to the parent compound, in conjunction with the formation of lower molecular weight cracking products. Conversion was measured in triplicate for each material and temperature, and the average error was less than 1% for all tested conditions.

Product selectivity was determined on a molar basis by normalizing the yield of each hydrocarbon fraction to the total number of moles of converted products. The hydrocarbons were subsequently classified into fuel-range groups according to their carbon numbers: C_3_–C_4_, light hydrocarbons; C_5_–C_8_, gasoline range; C_9_–C_12_, jet fuel-range fractions.

The catalyst deactivation constants (*k*_d_) were obtained from the slope of the linear regression of conversion versus time on stream, which is consistent with the approximately linear decay observed during the stability tests. The conversion decay was modeled using the following eq 1:

$$X(t)=X_{o}-k_{d}t$$ 1

where *k*_d_ denotes the slope of the fitted line. Conversion data were collected during continuous operation and a linear fit was performed for each catalyst.

## 3 Results and Discussion

### 3.1 Synthesis and Structural-Textural Properties of Surfactant-Templated HUSY-Based Zeolites

Surfactant-templated zeolites were synthesized following the procedure detailed in the methods section. The XRD analysis confirmed that the treated materials retained the characteristic diffraction patterns of their parent HUSY zeolites (Figure S1), with no additional reflections indicative of impurity phases or structural degradation. Specifically, the peak positions and intensities remained largely unchanged across all samples (CBV780_3h, CBV780_20h, CBV720_3h, and CBV720_41h), indicating that the FAU crystalline structure was preserved during the treatment. While the characteristic diffraction pattern was preserved, a modest reduction in the intensity of the reflections was observed, which aligns with previous reports showing that the introduction of mesoporosity typically leads to a decrease in diffracted intensity.^28^ For completeness, the relative decrease in diffracted intensity was quantified assuming the parent materials possessed 100% crystallinity, and the corresponding values are provided in the Supporting Information (Table S2).

The porous properties of the catalysts were investigated to assess the effects of surfactant templating on their micropore and mesopore volumes. Figure 1A,B show the nitrogen adsorption isotherms of both Pt/CBV780 and Pt/CBV720 series, and Table 1 summarizes the corresponding micro- and mesopore volumes. As expected, surfactant templating resulted in a significant increase in mesoporosity. For CBV780, the mesopore volume increased by 40% after 3 h of treatment and 147% after 20 h. A similar trend was observed for CBV720, which exhibited a 60% increase in mesopore volume after the surfactant-templating treatment. As illustrated in the pore size distribution profiles of all materials (Figure 1C,D), the surfactant-templating process results in the formation of mesopores with an average diameter of approximately 4.5 nm in both the CBV780 and CBV720 series. This pore size corresponded to the typical dimensions of micellar assemblies formed by cationic surfactants within the zeolite framework, indicating successful surfactant incorporation and mesostructural development.^13^

**Table 1:** Physicochemical Properties of the Studied Materials

		pore volume (cm^3^/g)^b^						
catalysts	Si/Al^a^	*V*_micro_	*V*_meso_ ^g^	**Bro̷nsted acid site density** ****(μmol/g**_**cat**_ **)** ^c^**	*d*_CO_ (nm)^d^	*d*_TEM_ (nm)^e^	Pt dispersion_CO_ (%)	*n*_Pt_/n_BAS_ ^f^
CBV780_Parent	40.0	0.24	0.23	139.8	6.2	14 (±8)	18.4	0.068
CBV780_3h	37.0	0.21	0.33	133.5	10.6	16 (±8)	10.7	0.041
CBV780_20h	37.9	0.18	0.58	147.5	4.3	11 (±8)	26.2	0.091
CBV720_Parent	15.0	0.26	0.22	332.2	3.2	6 (±4)	35.0	0.054
CBV720_3h	13.4	0.24	0.27	358.3	4.0	6 (±2)	28.2	0.040
CBV720_41h	12.2	0.19	0.35	350.7	1.5	7 (±5)	74.3	0.109

a Estimated with EDX prior to Pt-impregnation.

b Pore volumes obtained by nitrogen adsorption at 77 K using the NL-DFT method performed prior to Pt impregnation.

c Obtained by Py-FTIR.

d Obtained by CO chemisorption.

e Obtained by TEM.

f BAS = Bro̷nsted Acid Site.

g *V*_meso_ was estimated from 2 to 20 nm from the cumulative pore volume obtained using the NL-DFT method.

**Figure 1:**
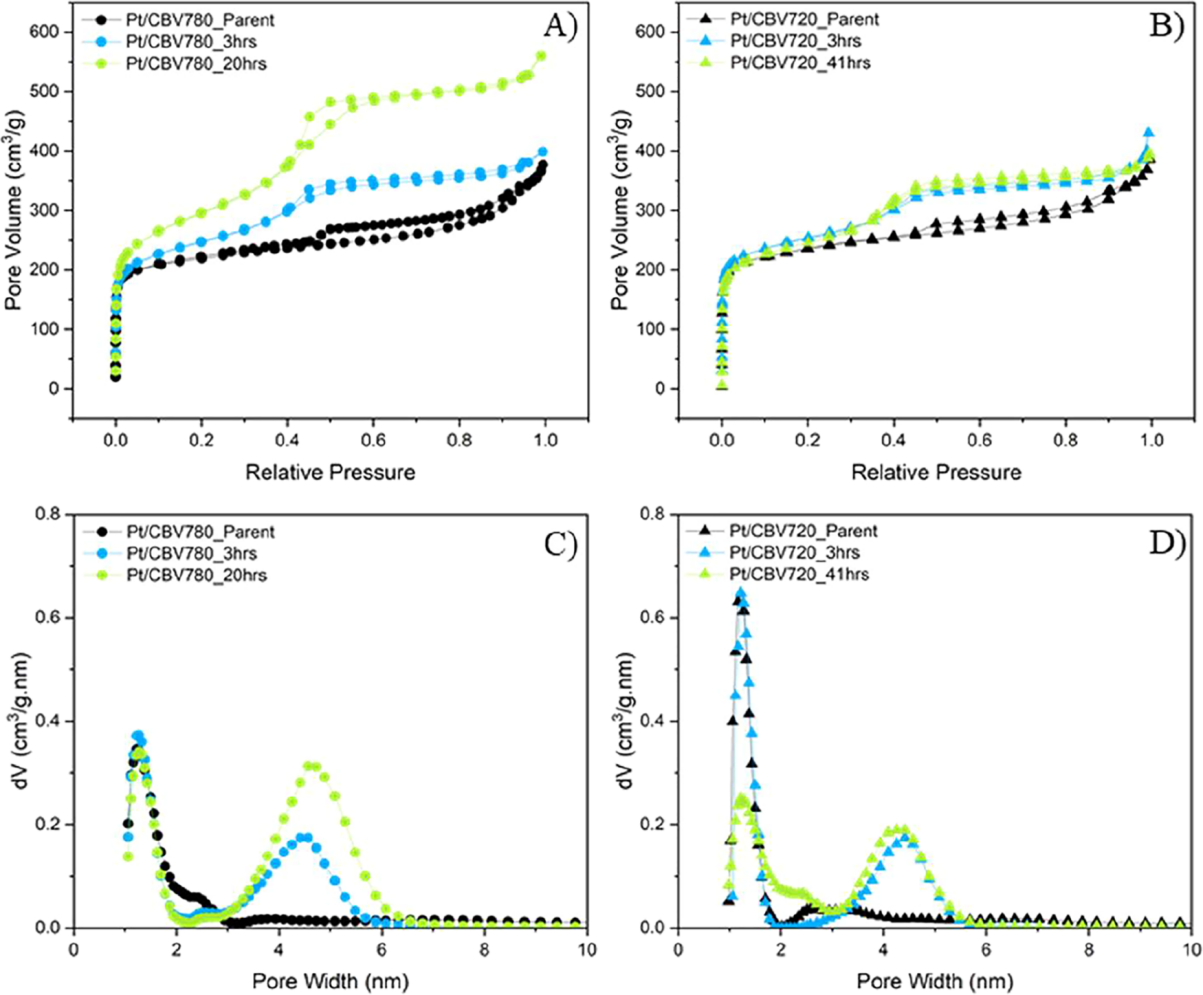
(A, B) Nitrogen adsorption isotherms and (C, D) pore size distributions of the zeolitic supports.

The superior mesoporosity observed for the CBV780 series arises from its high Si content. A higher Si/Al ratio increases the susceptibility of the framework to base-induced cleavage of Si–O–Al bonds, generating additional negatively charged Si–O^–^ sites that facilitate greater incorporation of the cationic surfactant into the zeolite. These sites play a crucial role in attracting the cationic surfactant CTAB into the zeolite interior. This interaction promotes micelle formation within the pores, ultimately leading to the development of mesopores. Consequently, CBV780 (Si/Al = 40) exhibited significantly greater mesopore formation than CBV720 (Si/Al = 15), under comparable treatment conditions.

While XRD confirmed the preservation of the long-range structural order, notable morphological transformations were evident, as illustrated in the micrographs in Figure 2. Figure 2A,D correspond to the parental zeolites, CBV720 and CBV780, respectively, both exhibiting large mesoporous channels, as indicated by the arrows. These features are attributed to the steaming process employed in their commercial preparation, which is often referred to as ultrastabilization.^7^ When the surfactant templating process was applied to the CBV720 sample after 3 h of treatment (Figure 2B), the formation of intracrystalline mesopores was evident, as highlighted by the circles. Extending the treatment to 41 h (Figure 2C) resulted in a more developed mesoporous network characterized by a higher density of well-defined, homogeneously distributed mesopores throughout the particle, indicating an advanced reconstruction of the internal framework. A similar trend was observed for the CBV780 series; after 3 h of surfactant templating (Figure 2E), the emergence of intracrystalline mesoporosity was apparent, although to a lesser extent compared to the CBV720 counterpart, once the mesoporous channels were still very visible. Prolonging the treatment to 20 h (Figure 2F) led to significant development of mesopores (circles), forming a pore network throughout the zeolite crystal. The combination of structural and porosity analyses clearly demonstrates that mesoporosity was successfully introduced into the zeolites, with minimal alteration to the crystalline framework. This integrated analysis confirms the formation of mesopores and highlights the structural integrity and mesostructural development throughout the zeolite crystals.

**Figure 2:**
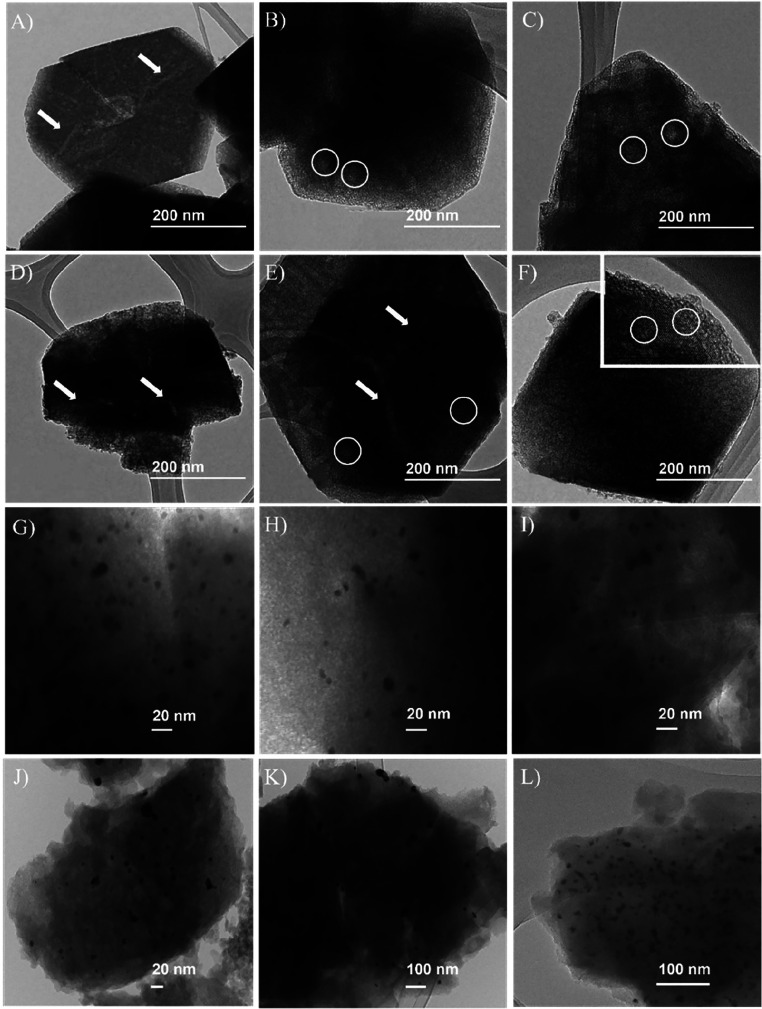
TEM images of (A) parent CBV720, (B) CBV720_3h, and (C) CBV720_41h zeolites. (D) Parent CBV780 zeolite, (E) CBV780_3h, and (F) CBV780_20h zeolites. White arrows indicate the presence of large mesoporous channels; the white circles highlight highly interconnected mesopores. High-magnification images of the samples after Pt deposition: (G) parent Pt/CBV720, (H) Pt/CBV720_3h, and (I) Pt/CBV720_20h samples; (J) Pt/CBV780 parent, (K) Pt/CBV780_3h, and (L) Pt/CBV780_20h samples.

All zeolites were subsequently used as supports for Pt immobilization to evaluate the enhanced diffusion properties imparted by the hierarchical structures. TEM micrographs (Figure 2G–L) revealed the presence of Pt nanoparticles (NPs) on both the parental and mesostructured zeolites. The particle sizes were determined from TEM images and corroborated by CO chemisorption measurements, and the results are summarized in Table 1. The corresponding particle size distribution histograms for all the catalysts are presented in Figure S2 (Supporting Information), further illustrating the TEM results. For the CBV780 series, the parent zeolite loaded with Pt (Figure 2G) shows NPs averaging 14 ± 8 nm (6.2 nm by CO). Using the 3 h surfactant-templating treated zeolite for metal immobilization (Figure 2H), the average particle size slightly increases to 16 ± 8 nm (10.6 nm by CO). However, using the zeolite with an extended treatment of 20 h (Figure 2I) resulted in a reduced particle size of 11 ± 8 nm (4.3 nm by CO), indicating that a more developed mesoporous structure facilitated improved control over the NPs’ size. In the CBV720 series, the parent zeolite (Figure 2J) contains smaller NPs, measuring 6 ± 4 nm (3.2 nm by CO). After 3 h of treatment (Figure 2K), the particle size remains essentially unchanged at 6 ± 2 nm (4.0 nm by CO). Prolonging the treatment to 41 h (Figure 2L) yielded the smallest NPs observed, approximately 7 ± 5 nm (1.5 nm by CO), suggesting that the mesoporous network formed in this series is particularly effective at stabilizing very small Pt NPs.

The evolution of Pt dispersion with increasing treatment time reflects the progressive structural modifications induced by the surfactant-templating process. As mesoporosity develops, the framework connectivity and the accessibility of internal acid sites increase, facilitating more effective anchoring of metal species. At short treatment times (3 h), both samples exhibit a slight decrease in Pt dispersion. This suggests that the framework is only partially reorganized at this stage, generating regions of local disorder and temporarily limiting access to stable anchoring sites. Under these conditions, Pt precursors may agglomerate during deposition, resulting in lower apparent dispersion. With prolonged treatment (20–41 h), the framework undergoes more extensive rearrangement and the micelles become fully organized within the crystal, yielding a well-connected mesopore network. This enhanced porosity improves mass transport and provides greater access to internal acid sites, enabling more uniform distribution and anchoring of Pt species. As a consequence, a marked increase in metal dispersion is observed at longer treatment times. The Si/Al ratio further modulates this behavior. Zeolites with higher Al content (lower Si/Al) possess a greater density of negatively charged framework sites capable of stabilizing metal cations, thereby promoting higher dispersion when the pore network is accessible.^29,30^ Furthermore, Pt-loading was determined using energy-dispersive X-ray spectroscopy, and the results are presented in Table S3. Following the characterization of the materials after the Pt loading (Table 1), the data revealed significant differences in physicochemical properties between the CBV780 and CBV720 series as a result of surfactant-templating treatment. Starting with the Si/Al ratio, the CBV780 series exhibited a high initial ratio of approximately 40 with only minor reductions after the surfactant-templating treatments, indicating that the framework remained largely stable with minimal dealumination or alteration. In contrast, the CBV720 series started with a Si/Al ratio of 15, which further decreased to 12.2 after 41 h of treatment. As expected, the high-aluminum materials exhibited acid site concentrations more than twice those of their low-aluminum counterparts, which is consistent with the values reported in the literature.^31,32^ More specifically, in terms of Bro̷nsted acidity, the CBV780 series maintained relatively low acid site densities throughout the treatments, with only a slight variation from 139.8 to 147.5 μmol/g. These results are consistent with the high Si/Al ratio and the low intrinsic acidity of this material. In contrast, the CBV720 series consistently displayed high Bro̷nsted acid site densities ranging from 332.2 to 350.7 μmol/g. Notably, the acid site density increased slightly with the templating process, suggesting that the introduction of mesoporosity did not compromise the acidic properties of the zeolite but rather preserved or even slightly enhanced them. These observations are further supported by the NH_3_-TPD profiles, which show no meaningful changes in peak position or intensity, confirming that the surfactant-templating treatment preserved both the strength and overall concentration of acid sites (Figure S3).

In addition to the Bro̷nsted acidity trends, pyridine-FTIR analysis revealed modest changes in the Lewis acid sites (LAS) after surfactant templating (Table S4). For the CBV780 series, the LAS values remained essentially constant within the experimental uncertainty, whereas the CBV720 series displayed a gradual increase with longer treatment times. Because the formation of EFAL-derived LAS typically requires the removal of framework Al, which would necessarily reduce BAS, the constant Bro̷nsted acidity observed for both series indicates that only limited EFAL formation or redistribution occurred during hydrothermal treatment. This interpretation is supported by the NH_3_-TPD results, which showed no significant changes in the acid site strength after templating. Given that hydrocracking proceeds predominantly on Bro̷nsted acid sites and that LAS contributes only secondarily, these modest LAS variations are not expected to meaningfully affect the catalytic behavior.

Another critical parameter for understanding the catalyst behavior, such as conversion, product selectivity, and stability, is the ratio of Pt active sites (number of surface-accessible Pt atoms) to Bro̷nsted acid sites (*n*_Pt_/*n*_BAS_). In the studied reaction system, olefins, which are known precursors of coke, contributed significantly to catalyst deactivation through the formation of carbonaceous deposits on the active surfaces. To preserve the catalyst stability and minimize coke formation, the *n*_Pt_/*n*_BAS_ ratio must exceed 0.03.^33,34^ Above this threshold, Pt sites hydrogenate olefinic intermediates rapidly enough to prevent coke build-up. Both Pt/CBV780 and Pt/CBV720 met this requirement (Table 1), indicating that their Pt loadings were sufficient to avoid premature deactivation.

Although some variations in nPt/nBAS were observed (Table 1), all catalysts remained well above the bifunctional regime in which hydrogenation–dehydrogenation steps were sufficiently fast to not limit the overall rate. This design choice ensured that Pt availability did not influence the observed trends, allowing the impact of mesoporosity and acid-site accessibility to be evaluated independently. Under these conditions, the rate-determining steps occur on the Bro̷nsted acid sites, so differences in hydrocracking activity and selectivity primarily reflect the acidic and textural properties of the hierarchical zeolites rather than variations in Pt content.

### 3.2 Cracking Activity Evaluation

The catalytic performance of all Pt-supported HUSY zeolites (both parent and mesostructured) was evaluated in the hydrocracking of n-hexadecane as a function of the reaction temperature and mesoporosity development. The catalytic performances of the Pt/CBV780 and Pt/CBV720 series catalysts was strongly influenced by the structural and textural modifications induced by the surfactant-templating treatment (Figure 3). For the CBV780 series (Figure 3A), Pt/CBV780_3h showed modest conversion improvements of 4% at 350 °C over the conventional catalyst (Pt/CBV780), as expected owing to the small amount of mesoporosity introduced; however, the most significant enhancement was observed for Pt/CBV780_20h, delivering an 11% increase in conversion at the same temperature. The large amount of interconnected mesoporosity in the sample treated for 20 h also led to higher conversions at lower temperatures, exhibiting gains of 4, 6, and 12% at 275, 300, and 325 °C, respectively, which confirms that the diffusion constraints were greatly alleviated, considering that the total number of Bro̷nsted acid sites was the same between the samples and the *n*_Pt_/*n*_BAS_ was high enough that the acid function governed the reaction.

**Figure 3:**
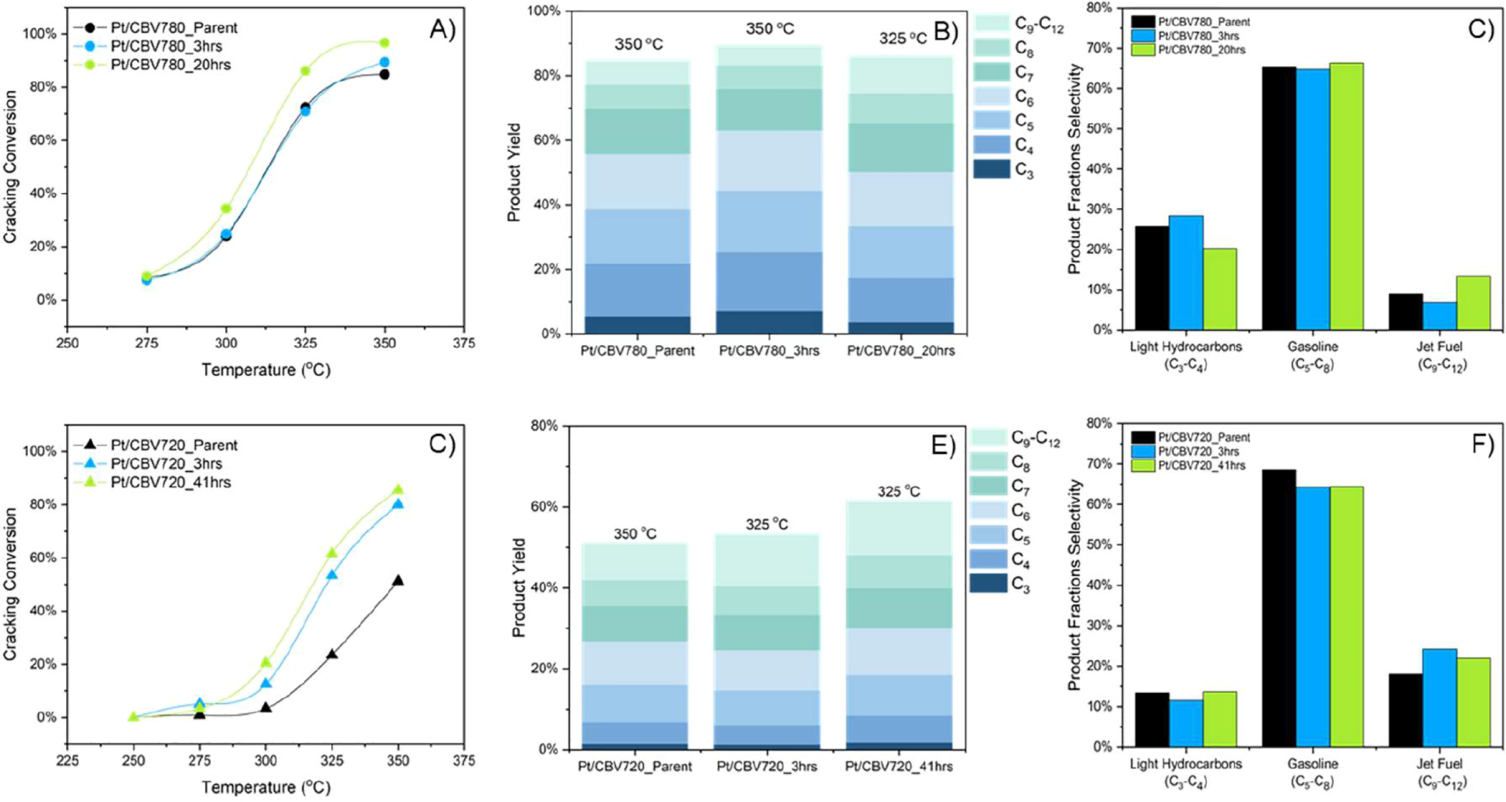
(A, D) *n*-Hexadecane hydrocracking conversion as a function of reaction temperature, (B, E) product yields at isoconversions, and (C, F) product fraction distributions at isoconversions over nontreated and surfactant-templated Pt/CBV780 (A–C) and Pt/CBV720 (D–F) catalysts. Reaction conditions: 0.03 mL hexadecane/min, 30 mL/min of H_2_, and 20 bar of pressure. Carbon balance: 99.8 ± 2.1%.

An increased effect was observed in the CBV720 series (Figure 3D). In this case, the catalyst containing a modest amount of surfactant-templated mesoporosity (Pt/CBV720_3h) already exhibited a 29% increase in conversion at 350 °C compared to the conventional Pt/CBV720. Notably, the sample with more mesoporosity (Pt/CBV720_41h) achieved a remarkable 34% increase in conversion at the same temperature. These conversion gains were also observed in Ni/H-Beta catalysts when intracrystalline mesoporosity was introduced, increasing *n*-hexadecane conversion by approximately 31%, attributing this enhancement to improved accessibility of acid sites and reduced diffusion limitations.^25^

A key finding emerged when comparing the product yields of the surfactant-treated samples at isoconversion, that is, at 80% conversion for the CBV780 series (325 °C for Pt/CBV780_20h and 350 °C for Pt/CBV780_Parent and Pt/CBV780_3h) and 50% conversion for the CBV720 series (325 °C for Pt/CBV720_3h and Pt/CBV720_41h and 350 °C for Pt/CBV720_Parent) (Figure 3B,E). Typically, higher temperatures promote increased cracking, favoring the formation of lighter hydrocarbons owing to enhanced thermal energy driving bond scission. However, at isoconversion, the product distribution of the surfactant-treated samples at 325 °C closely resembled that of the parent material at 350 °C. Notably, and despite the lower reaction temperature, the mesoporosity introduced by the surfactant treatment resulted in the formation of C_9_–C_12_ products in greater proportions (with yield gains of 5% for the Pt/CBV780 series and 4% for the Pt/CBV720 series), while maintaining similar C_5_–C_8_-range product selectivity (Figure 3C,F). This enhanced selectivity toward heavier products is attributed to the reduced transport constraints that the C_9+_ intermediate experiences within the mesoporous structure, inducing a higher degree of outward diffusion rates rather than consecutive cracking transformations.^35,36^

Comparable results have been reported elsewhere, showing that increasing the mesoporous fraction in hybrid micro-mesoporous HY catalysts enhances selectivity toward gasoline-jet-fuel hydrocarbons while simultaneously suppressing the formation of light gases, accompanied by higher *n*-hexadecane conversion.^23^ Similar effects in hierarchical Ni/Des-H-Beta catalysts showed preferential formation of C_5_–C_10_ cracking products attributed to the same accelerated transport of reactive intermediates out of the pore system, reducing the likelihood of consecutive isomerization or recracking steps.^25^

This reinforces the enhanced molecular diffusion owing to the presence of the mesoporous structure of the surfactant-templated sample, which plays a crucial role in directing product selectivity and mitigating the need for higher temperatures to achieve the same degree of cracking efficiency. A noteworthy observation is the absence of olefins among the products during the gas and liquid phase product analysis using mass spectroscopy, demonstrating the effectiveness of platinum in hydrogenating unsaturated species formed during C–C bond scission. This limits the reabsorption of unsaturated intermediates for further reactions, thereby nearly fully inhibiting secondary reactions that could lead to coke formation.

The improvement in the catalytic conversion per unit of acid sites was also analyzed as a function of mesopore volume (Figure 4). In the Pt/CBV780 series, increased mesopore volume modestly enhanced the catalytic conversion of hexadecane, with improvements of 13% at 325 °C and 8% at 350 °C relative to the parent catalyst. In contrast, the Pt/CBV720 series demonstrated substantially greater sensitivity to mesopore development. The expanded mesopore network led to turnover rate increase of 126% at 325 °C and 58% at 350 °C compared with the parent material.

**Figure 4:**
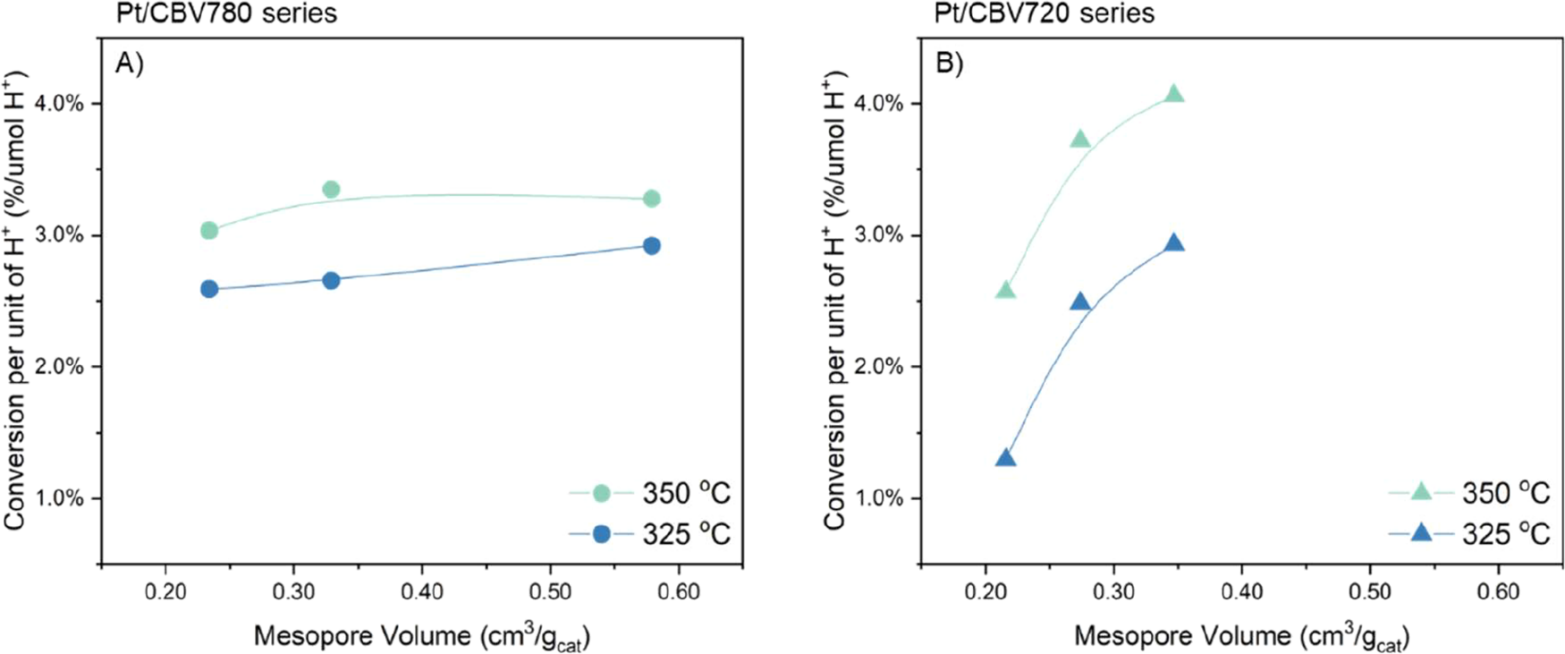
Catalytic cracking conversion per unit of Bro̷nsted acid sites comparison on the catalysts in (A) Pt/CBV780 and (B) Pt/CBV720 series. Reaction conditions: 0.03 mL hexadecane/min, 30 mL/min of H_2_, and 20 bar of pressure.

The differences in the catalytic performance can be partly explained by the difference in the concentration of acid sites present in the Pt/CBV780 and Pt/CBV720 parent zeolites. Although both parent catalysts had comparable mesoporosity, the higher acid site density in Pt/CBV720_Parent created a greater need for higher diffusion rates to maintain the proper concentration of reactant molecules in the internal sites of the catalyst. As a result, Pt/CBV720_Parent inherits diffusion limitations that negatively affect the conversion rate, whereas Pt/CBV780_Parent, with a lower acid site density, has fewer limitations in this regard. The introduction of additional mesoporosity in Pt/CBV720_Parent improves the diffusion of larger molecules such as n-hexadecane, leading to a significant increase in the local reactant concentration around the reaction site, thus improving the reaction rate. This highlights that while mesoporosity helps improve accessibility and diffusion, acid site density and diffusion limitations are determining factors in the final catalytic performance, explaining the differences in the first observed intrinsic rate. Moreover, the introduction of additional mesopores had a more pronounced effect on improving the catalytic performance of CBV720 than CBV780. Moreover, the spatial distribution of Al atoms, particularly the prevalence of Next-Nearest-Neighbor (NNN) Al pairs, differed significantly between the two zeolites. In CBV720, the higher framework Al content increases the probability of Al pairs occupying NNN positions, which facilitates the formation of Bro̷nsted acid sites with a higher local proton density.^37,38^ This arrangement promotes cooperative acid-site interactions and enhances the cracking of long-chain hydrocarbons.

### 3.3 Stability Assessment of *n*-Hexadecane Cracking Performance

As a result of the intracrystalline mesoporosity, surfactant-templated zeolites provide shorter diffusion path lengths, which enhance hydrocracking performance and diminish the occurrence of secondary coke-forming reactions. To gain deeper insight into this phenomenon, the conversion at 350 °C was monitored for all samples studied. Experimentally, the two series were subjected to different time-on-stream durations, and the CBV780 series was evaluated under the reaction conditions for approximately 3 days, whereas the CBV720 series underwent stability testing for 11 days. This discrepancy stems from the distinct catalytic behavior of each material, which necessitates different operational windows and ultimately contributes to different coke formation levels. Despite starting at different initial conversion levels, the surfactant-treated catalysts demonstrated improved stability compared with that of the parent zeolite (Figure 5A,B).

**Figure 5:**
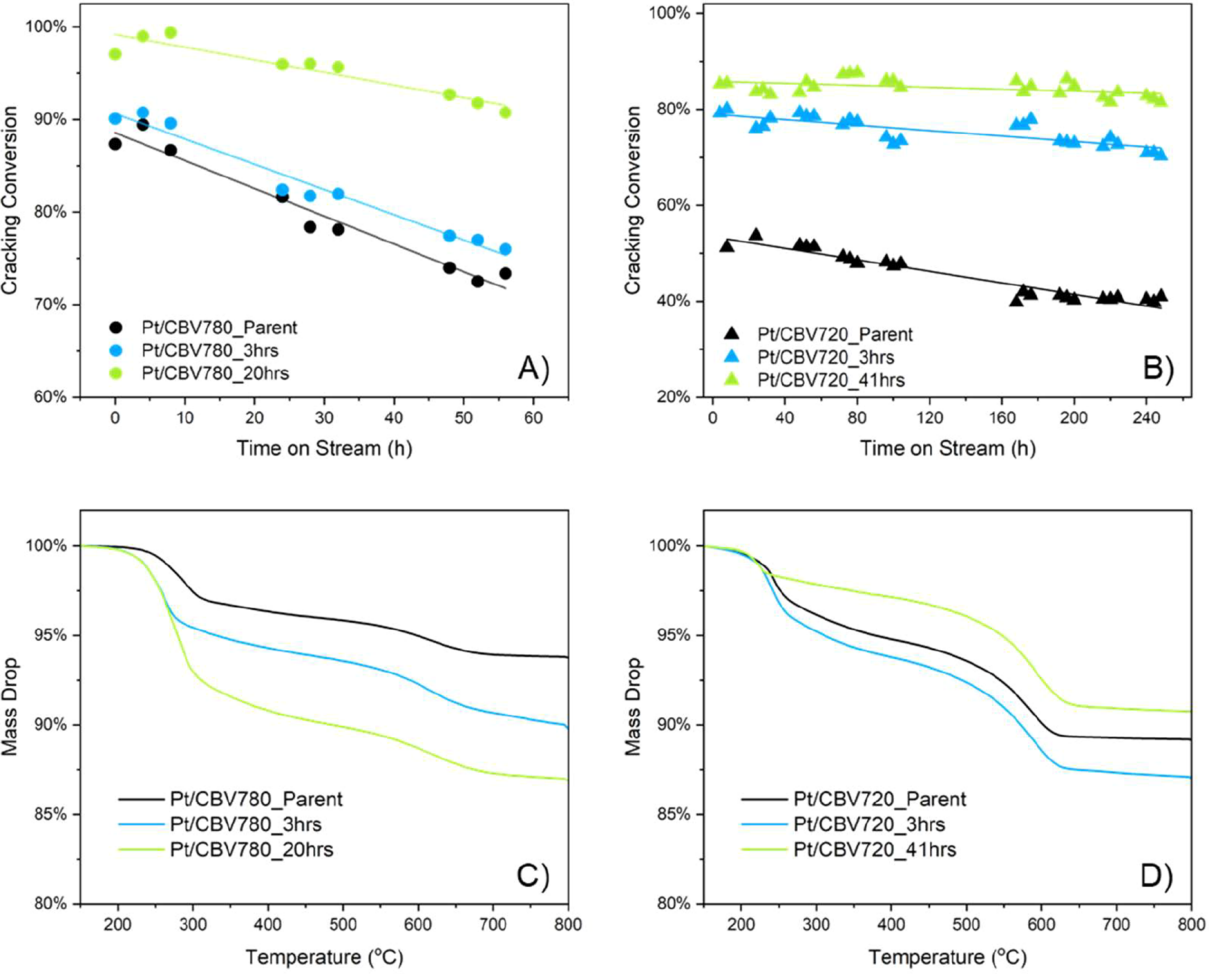
Cracking conversion evolution as a function of time on stream for the (A) CBV780 and (B) CBV720 series. Reaction conditions: 350 °C, 0.03 mL hexadecane/min reactant feeding, and 20 bar of H_2_. TG of postreaction catalysts: (C) Pt/CBV780 series after 3 day stability testing and (D) Pt/CBV720 series after 11 day stability testing.

The catalysts exhibited a linear decrease in conversion with time on stream, with the untreated sample showing the fastest loss of activity and the surfactant-templated catalysts displaying markedly improved stability. Deactivation constants were obtained by fitting the conversion data to a linear rate equation over time (Table 2). The untreated catalyst showed a high deactivation constant (*k*_d_) consistent with the steep decline in activity, whereas the surfactant-templated materials presented substantially lower *k*_d_ values, indicating a more gradual deactivation rate. This behavior agrees with trends reported in the literature for stabilized catalysts during deactivation–regeneration cycles in VGO cracking.^39^

**Table 2:** TG Results on Post-Reaction Catalysts Tests and Calculated Deactivation Constants of Nontreated and Surfactant-Treated USY

			*d*_TEM_ (nm)	
catalyst	coke_400C_ (m/m)	*k*_d_ (*X*_hexadecane_ × 10^–4^ h^–1^)^a^	before	after
Pt/CBV780_Parent	2.6%	31.4	14 ± 8	13 ± 7
Pt/CBV780_3h	4.5%	27.3	16 ± 8	14 ± 7
Pt/CBV780_20h	4.1%	13.6	11 ± 8	10 ± 4
Pt/CBV720_Parent	6.1%	6.2	6 ± 4	5 ± 2
Pt/CBV720_3h	7.2%	2.8	6 ± 2	7 ± 3
Pt/CBV720_41h	6.5%	1.3	7 ± 5	5 ± 2

a Calculated on a linear basis conversion profile.

In the CBV720 catalyst series, consistent stability was observed throughout the reaction period, and the nPt/nBAS ratios were similar to those of the CBV780 series. As previously discussed, the superior performance of the CBV720-based catalysts can be attributed to the synergistic effects arising from the spatial proximity between the metal and acid sites, which contributes to the maintenance of an active catalytic surface. Although the parent CBV720 sample displayed a lower initial activity and a gradual decline in conversion, likely owing to the diffusion limitations inherent to its microporous structure, the surfactant-treated samples benefited from the enhanced accessibility, resulting in improved stability.

The coke deposition increased with longer surfactant treatment times (Table 2). Although enhanced intracrystalline diffusion typically reduces the residence time of coke precursors and thus limits coke formation, greater coke accumulation was observed. This can be explained by the higher conversions observed for the surfactant-templated samples and the increased pore volumes of the coke species. In other words, these catalysts are processed and converted a greater amount of feed than the parent material, naturally generating more coke in absolute terms without indicating a lower stability. TG (Figure 5C,D) suggested that most of the coke was derived from lighter species, as indicated by the sharp mass losses at lower temperatures, whereas a smaller contribution at higher temperatures was associated with bulky, more complex compounds. This trend was attributed to the higher intrinsic activity of the mesoporous samples, which facilitated secondary transformations and polymerization reactions, leading to increased coke formation.^39^ Nonetheless, the mesoporous catalysts retained more accessible acid sites and exhibited longer catalytic lifetimes than their parent counterparts, even at higher coke content.^40^ Therefore, despite the greater coke deposition observed on the surfactant-treated catalysts, their improved diffusion properties and preserved acidity contribute to their sustained catalytic activity over extended operation.

Another important factor is the accessibility of sites for the physical deposition of carbon species, particularly in the Pt/CBV780 samples, as evidenced by the TG results. The largest mass losses occur at lower temperatures and are typically associated with the physical or chemical adsorption of lighter compounds, which are difficult to remove except under specific conditions. Complete or partial combustion of these materials is unlikely at these temperatures.^41,42^ Therefore, a significant portion of the internal region of the catalyst is accessible for the deposition of lighter carbonaceous materials.

TEM imaging of the spent catalysts confirmed that the Pt nanoparticles remained stable across all the materials after exposure to the reaction conditions (Table 2 and Figure S3). The reaction temperature and hydrogen-rich environment did not induce significant Pt sintering or agglomeration. Importantly, the introduction of mesoporosity did not affect the intrinsic stability of the Pt particles, which was primarily attributed to the spatial confinement imposed by the restricted dimensions of the zeolite intracrystalline cavities, which limited particle migration and growth. This finding aligns with previous reports and further reinforces the conclusion that mesoporosity not only enhances catalytic activity, but also contributes positively to catalyst stability by facilitating mass transport without compromising metal dispersion.^43,44^

## 4 Conclusions

Overall, the surfactant-assisted modification of Pt/HUSY catalysts proved highly effective in enhancing both the catalytic performance and stability in the hydrocracking of n-hexadecane, emphasizing the advantages of this controlled mesostructuring route over conventional treatments. The surfactant-mediated approach generated a substantial increase in mesopore volume, approximately 2.5-fold for the CBV780 series and 1.6-fold for the CBV720 series, while preserving the key physicochemical features of the parent zeolites, including crystallinity and the concentration of Bro̷nsted acid sites. Notably, the higher-silica zeolite (CBV780) exhibited a greater propensity for mesoporosity development under milder conditions, whereas the lower-silica CBV720 required more severe treatment to achieve comparable textural modifications.

The influence of mesoporosity on catalytic performance was evident, with an overall conversion increase of 12–36% relative to the parent zeolites. When normalized per Bro̷nsted acid site, the activity trends revealed distinct diffusion effects between the two series of catalysts. For the CBV780-based catalysts, only moderate improvements were observed, suggesting that the parent material was not strongly limited by the internal diffusion of long-chain *n*-hexadecane. In contrast, the CBV720-based catalysts exhibited significant activity enhancements, as the introduction of mesoporosity effectively mitigated the severe transport limitations that had previously hindered the full utilization of the acid sites. Despite this, CBV720 maintained intrinsically higher activity than CBV780, likely because of the closer proximity of its acid sites. This enhanced accessibility within the mesostructured materials promoted deeper cracking, favoring the formation of lighter hydrocarbons at a given temperature at the expense of heavier intermediates within the C_9_–C_12_ range products.

Finally, the mesoporous catalysts demonstrated improved stability compared to their parent counterparts. The lower deactivation constants and smoother conversion decay profiles indicated that the treated zeolites retained higher activity over extended operation, even though slightly greater coke accumulation was detected. Collectively, these findings establish a direct correlation between the mesopore development, diffusion efficiency, and catalytic stability. They confirmed that surfactant-assisted mesostructuring effectively enhanced the performance of Pt/HUSY catalysts by improving the accessibility to active sites, tuning product selectivity, and providing valuable design principles for next-generation hierarchical zeolitic catalysts in hydrocarbon conversion processes.

## Supplementary Material



## Notes

Detailed information on the catalyst mass, hexadecane flow rates, and W/F values employed in the catalytic tests; relative crystallinity of both parent and surfactant-templated zeolites, as determined by XRD, along with the elemental composition of the Pt/CBV780 and Pt/CBV720 catalysts, as analyzed by EDX; pyridine-FTIR quantification of Bro̷nsted and Lewis acid sites for all Pt/HUSY catalysts; X-ray diffraction patterns of the studied zeolites, with or without surfactant treatment; particle size distributions of Pt nanoparticles on surfactant-templated and parent USY supports; ammonia temperature-programmed desorption (NH_3_-TPD) profiles of the zeolite samples; and TEM images of spent materials, including those of parental CBV780_Parent, CBV780_3h, CBV780_20h, CBV720_Parent, CBV720_3h, and CBV720_41h zeolites (PDF)
