# Supplementary material for: Enhancing Hydrocracking Catalyst Performance and Lifetime through Surfactant-Templated Mesoporosity in Pt/HUSY Zeolites
Source: Energy Fuels. 2025 Dec 27;40(1):730–41. doi: 10.1021/acs.energyfuels.5c05175 (PMC12797223; doi:10.1021/acs.energyfuels.5c05175)
Supplement: Supplementary file 1 [file ef5c05175_si_001.pdf]

Supporting Information

Enhancing Hydrocracking Catalyst Performance and Lifetime through Surfactant-Templated Mesoporosity in Pt/HUSY Zeolites

N.F.L. de Paula[1], H.M. Mesa[1], J.M. Ortigosa[2], M.A.S. Garcia,[3] J.M.A.R. de Almeida[1,4], J. Garcia-Martinez[2,*], P.N. Romano[1,3,4,*]

[1]Instituto de Química, Universidade Federal do Rio de Janeiro, Av. Athos da Silveira Ramos, 149, Rio de Janeiro, Brazil 21941-909.

[2]Laboratorio de Nanotecnología Molecular, Departamento de Química Inorgánica, Universidad de Alicante, 03690, Alicante, Spain.

[3]Nanotechnology Engineering Program, Alberto Luiz Coimbra Institute for Graduate Studies and Research in Engineering, COPPE, UFRJ, Rio de Janeiro 21941-972, RJ, Brazil.

[4]Programa de Pós-Graduação em Química (PGQu), UFRJ, Rio de Janeiro 21941-909, RJ, Brazil

*pedroromano@iq.ufrj.br

*j.garcia@ua.es

Catalyst Masses, Bed Configuration, and W/F Values Used in Catalytic Tests

To accurately define the experimental conditions used in the catalytic testing, Table S1 reports the catalyst masses, hexadecane flow rates, and corresponding W/F values for each reaction run. Because the CBV720 samples required reduced catalyst masses to achieve equivalence in Brønsted acid site density, they were diluted with inert glass beads to match the packed-bed height and void fraction of the CBV780 reactor bed. This procedure ensured comparable hydrodynamic behavior and, therefore, consistent residence times across all experiments, irrespective of the catalyst series. The reported values correspond precisely to the conditions under which all kinetic measurements were obtained.

Table S1. Catalyst mass, total inlet flow rate, and corresponding W/F values used in the catalytic tests.

**Base Catalyst**	**Catalyst mass (mg)**	**Hexadecane flow (mL/min)**	**W/F ($g_{catalyst}$.min.$g_{hexadecane}^{-1}$)**
CBV780	200	0.03	8.66
CBV720	60	0.03	2.60

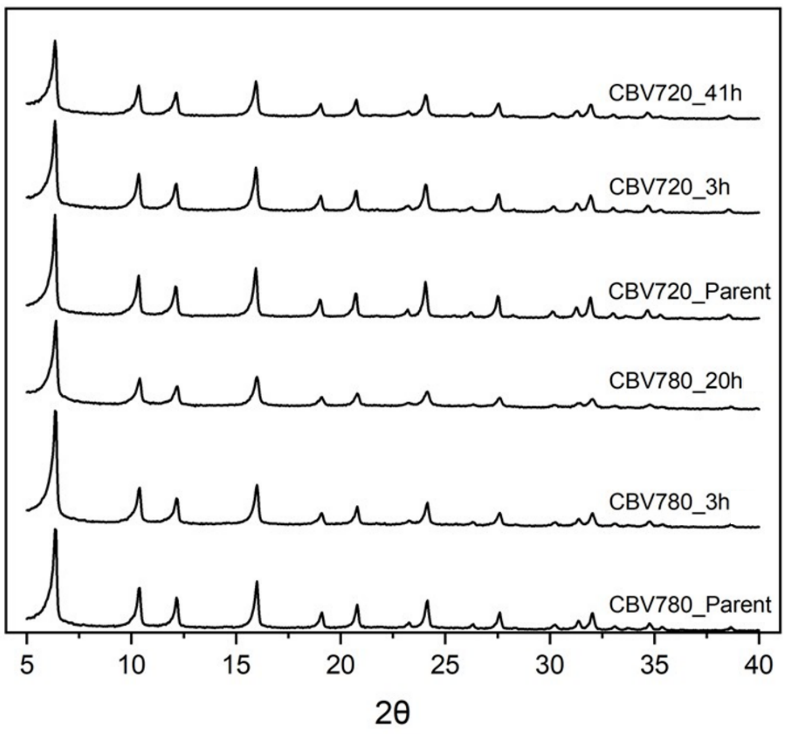

Figure S1. XRD of the studied zeolites, with or without treatments.

Quantification of Relative Crystallinity from XRD Patterns

To evaluate the impact of the surfactant-templating treatment on the structural integrity of the zeolite framework, the relative crystallinity of all samples was quantified from their X-ray diffraction (XRD) patterns. Although the characteristic FAU reflections were preserved in all materials, a slight reduction in diffraction intensity was observed, consistent with the expected framework rearrangements that accompany mesopore formation.

Relative crystallinity was calculated by integrating the area under the diffraction peaks within the range of 8–40° (2θ), corresponding to the high-angle region associated with the ordered microporous framework of FAU-type zeolites. The integrated intensities of the treated samples were normalized to those of the corresponding parent zeolites (CBV780 and CBV720), which were assigned a value of 100% crystallinity. This approach allows the quantification of the decreases in signal arising from mesopore insertion, which reflect partial redistribution of framework domains rather than structural degradation. The resulting crystallinity values for all samples (CBV780_3h, CBV780_20h, CBV720_3h, and CBV720_41h) are compiled in Table S2.

Table S2. Relative crystallinity of parent and surfactant-templated zeolites.

**Sample**	**Crystallinity**
CBV780_Parent	100%
CBV780_3h	84%
CBV780_20h	77%
CBV720_Parent	100%
CBV720_3h	93%
CBV720_41h	73%

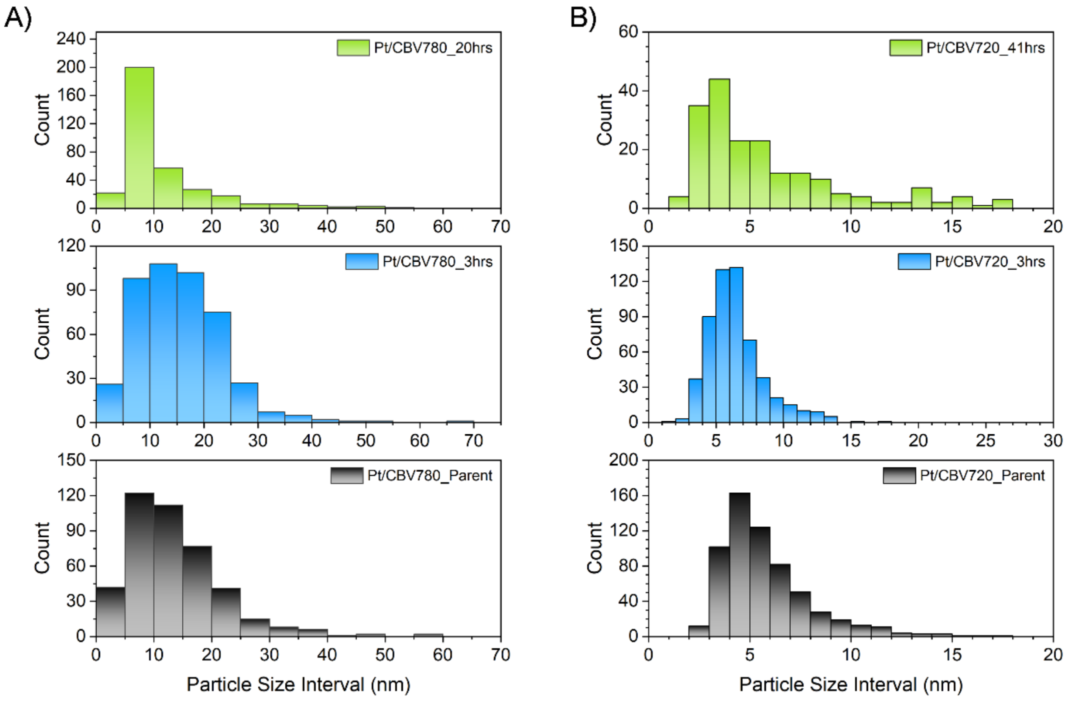

Figure S2. Particle Size Distributions of Pt nanoparticles on surfactant-templated and parent USY Supports.

Elemental Composition by Energy Dispersive X-ray Spectroscopy of Pt-Loaded USY Catalysts

The EDX results confirm that the surfactant-templating treatments do not significantly alter the Pt loading in either the CBV780 or CBV720 series, as the measured Pt wt.% remains within a narrow range for both parent and treated samples. These findings indicate that the hierarchical structuring process does not compromise the amount of metal incorporated into the catalysts.

Table S3. Elemental composition of Pt/CBV780 and Pt/CBV720 catalysts determined by EDX.

	**EDX (wt.%)**					
	**Pt/CBV780**			**Pt/CBV720_Parent**		
	**Parent**	**3hr**	**20hr**	**Parent**	**3hr**	**41hr**
Si	41%	46%	51%	55%	53%	47%
O	54%	52%	47%	41%	43%	47%
Al	3%	1%	1%	3%	3%	4%
Pt	0.7%	0.7%	0.9%	2.0%	1.2%	1.6%

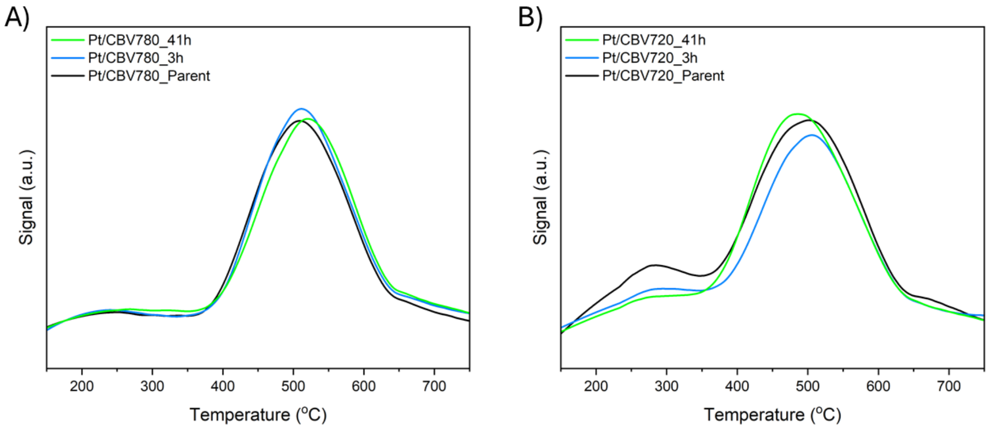

Figure S3. Ammonia Temperature-Programmed Desorption (NH3-TPD) profiles of zeolite samples; A) Pt/CBV780 and B) Pt/CBV720 series respectively.

Acidity Characterization: Brønsted–Lewis Site Quantification

The full numerical values of Lewis (LAS), Brønsted (BAS) and total acid sites assessed by pyridine FTIR are provided below. The data confirm that BAS densities remain essentially constant upon surfactant-templating, indicating minimal framework Al extraction. The modest increases in LAS, more pronounced in the CBV720 series, are consistent with minor EFAL redistribution during surfactant-treatment. These variations are small relative to the dominant Brønsted acidity and therefore have limited impact on hydrocracking performance.

Table S4. Pyridine-FTIR quantification of Brønsted and Lewis acid sites for all Pt/HUSY catalysts.

Samples	Lewis Acid sites ($\mu mol.g^{-1}$)	Bronsted Acid sites ($\mu mol.g^{-1}$)	Total Sites ($\mu mol.g^{-1}$)
Pt/CBV780_Parent	52	140	192
Pt/CBV780_3h	70	134	204
Pt/CBV780_20h	48	148	196
Pt/CBV720_Parent	101	332	433
Pt/CBV720_3h	146	358	504
Pt/CBV720_41h	193	322	515

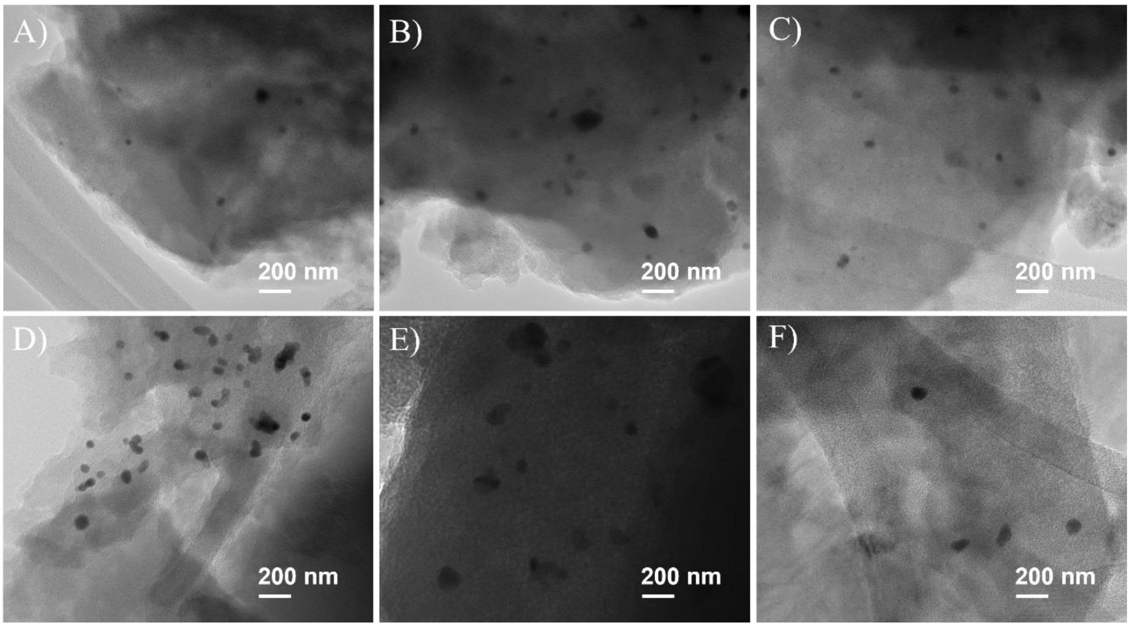

Figure S4. TEM images of spent materials. A) parental CBV720, B) CBV720_3h, and C) CBV720_20h zeolites. D) Parental CBV780 zeolite, E) CBV780_3h, and F) CBV780_20 h zeolites.
